# In Children and Adolescents (Ages 6-17) With Attention Deficit Hyperactivity Disorder (ADHD), How Does Viloxazine Extended-Release (ER) Compare with Placebo or Other ADHD Medications in Terms of Improving ADHD Symptoms, Adverse Events and Treatment Discontinuation Rates? A Systematic Review

**DOI:** 10.1192/bjo.2025.10198

**Published:** 2025-06-20

**Authors:** Khushboo Kansal, Betsy Marina Babu, Gaurav Uppal, Nadia Liaqat, Asha Dhandapani

**Affiliations:** ^1^Nottinghamshire NHS Trust, Nottingham, United Kingdom; ^2^London and KSS school of Psychiatry, London, United Kingdom; ^3^Satyam Hospital, Ludhiana, India; ^4^East London NHS, London, United Kingdom; ^5^BCUHB, Wrexham, United Kingdom

## Abstract

**Aims:** In this systematic review, the effectiveness and safety of viloxazine ER in the treatment of ADHD in children and adolescents aged between 6–17 years will be assessed.

**Methods:** This review identified articles through a systematic approach using PubMed, EMBASE, and the Cochrane Library. Randomized controlled trials with viloxazine ER in an active comparator condition versus placebo or other stimulant/non-stimulant ADHD drugs were included.

The first set of outcomes for assessing efficacy was a decrease in the severity of ADHD symptoms as measured by the ADHD-RS-5 and CGI-I scales. Safety outcomes comprised comparability in the rates of adverse events and treatment discontinuation rates.

**Results:** A meta-analysis showed that viloxazine ER is effective in managing ADHD symptoms compared with placebo at 10–12 weeks. Very few side effects were reported with this medication and those reported were mostly mild to moderate in nature. Mild side effects were noted to be decreased appetite, somnolence, and headache. The rates of treatment disappearance were similar compared with other oral ADHD drugs.

**Conclusion:** The research implies that viloxazine ER may be useful to paediatric patients with ADHD as a new treatment approach. We hypothesize that its profile of being an NRI and 5-HT2B antagonist may be beneficial for patients who have not shown sufficient improvement with more common treatments. The use of once daily dosing of the extended release formulation may enhance compliance compared with drugs taken more than once per day.

In a general manner, viloxazine ER seems to be a safe and efficacious therapy in children and adolescents affected by ADHD. Because it has a unique mechanism of action and can be taken once daily, it complements other ADHD medications well. More prospective, multicentre trials of longer duration are, therefore, required to determine the success and risks of the technique in the long run.

